# Side Effects of COVID-19 Vaccination Among Medical Students in Bangladesh: A Cross-Sectional Study

**DOI:** 10.7759/cureus.96937

**Published:** 2025-11-15

**Authors:** Abu Saleh Md Sadequl Islam, Sanjida Sanjana, Syed U Azad, Sadman Saify, Mustari Sarkar Trisha, Mahmudul Hasan Nahid

**Affiliations:** 1 Hepatology, Shaheed Ziaur Rahman Medical College Hospital, Bogura, BGD; 2 Medicine, Shaheed Ziaur Rahman Medical College Hospital, Bogura, BGD; 3 Medicine, City Medical College and Hospital, Gazipur, BGD; 4 Public Health, University of Nottingham, Nottingham, GBR; 5 Acute Internal Medicine, University Hospitals of Leicester, Leicester, GBR; 6 Internal Medicine, Shaheed Ziaur Rahman Medical College Hospital, Bogura, BGD

**Keywords:** adverse effects, bangladesh, covid-19 vaccines, long post-covid vaccination syndrome, medical students, reactogenicity, vaccine hesitancy, vaccine side effects

## Abstract

Background

Vaccination is a cornerstone of COVID-19 control, yet concerns about side effects continue to influence acceptance. Evidence from medical students in low- and middle-income countries is limited, despite their future role in guiding public attitudes.

Methods

A cross-sectional online survey was conducted among undergraduate medical students in Bangladesh between February 2021 and April 2022. Data on demographics, vaccination history, and post-vaccination symptoms were collected through a structured questionnaire. Descriptive statistics, chi-square tests, and multivariable Firth logistic regression were used to examine associations between sex, vaccine type, and side effects.

Results

Of 305 respondents (mean age = 21.6 years; 196 females, 64.3%), 293 (96.1%) and 286 (93.8%) received the first and second vaccine doses, respectively, while 191 (62.6%) received a third dose. The most common vaccines were Sinopharm (125 participants, 41.0%), Pfizer-BioNTech (68 participants, 22.3%), and Moderna (60 participants, 19.7%). Overall, 276 participants (90.5%) experienced at least one side effect, with the prevalence declining across doses: 270 (88.5%) after the first dose, 243 (79.7%) after the second dose, and 128 (42.0%) after the third dose. The most frequent complaints were local pain (508 cases, 41.2%), fever (184, 14.9%), rash or itch (114, 9.2%), headache (108, 8.8%), and myalgia (103, 8.3%). Less common outcomes included anxiety or low mood (70, 5.7%) and alopecia (45, 3.6%), while serious events were rare. Female participants had higher odds of experiencing second-dose side effects (OR: 1.85, 95% CI: 1.05-3.26). Compared with Moderna, Pfizer-BioNTech was associated with fewer third-dose events (OR: 0.45, 95% CI: 0.21-0.94).

Conclusion

COVID-19 vaccines were well-tolerated among Bangladeshi medical students, with most side effects mild and self-limiting. Reactogenicity declined with subsequent doses and varied by sex and vaccine type. These results emphasize the need for transparent communication and vaccine literacy within medical education to enhance students’ confidence and their role in addressing vaccine hesitancy.

## Introduction

The COVID-19 pandemic has dramatically reshaped the global landscape, causing widespread public health, economic, and social disruption. Since its emergence in December 2019, caused by the SARS-CoV-2 virus, the pandemic has had a profound impact worldwide, leading to over 774.8 million confirmed cases and 7.03 million deaths as of early 2024 [1]. The rapid spread of the virus led to an urgent global response, with countries implementing strict measures, including lockdowns, social distancing, and accelerated vaccine development. By March 2024, Bangladesh had reported over 2.04 million cases and 29,491 deaths, emphasizing the critical importance of strong public health strategies [2].

The development and distribution of vaccines have been central to controlling the pandemic. Vaccines such as Pfizer-BioNTech, Moderna, AstraZeneca-Oxford (Covishield), and Sinovac have played a pivotal role in mitigating the spread of the virus and reducing the severity of illness [3]. Bangladesh initiated its vaccination program in February 2021, prioritizing healthcare workers, elderly individuals, and high-risk populations before expanding to the general public [4]. Despite these achievements, challenges such as vaccine hesitancy, misinformation, and concerns about side effects continue to hinder global vaccination efforts. In Bangladesh, hesitancy has been particularly prevalent among younger adults and students, highlighting the importance of understanding the perceptions and experiences of this demographic [5].

Medical students, who will eventually play a significant role in healthcare delivery, are in a unique position to influence vaccine acceptance among the public. As a group that is scientifically literate and closely engaged with healthcare systems, their perceptions and experiences can provide valuable insights into vaccine hesitancy and reported side effects. However, the experiences of medical students regarding COVID-19 vaccination, particularly in low- and middle-income countries like Bangladesh, remain underexplored. Most existing research has focused on healthcare workers or the general population, with limited attention to medical students. Given their future role in shaping public attitudes, understanding medical students’ experiences and perceived side effects is essential.

The rapid rollout of COVID-19 vaccines has generated extensive data on their safety and efficacy. Studies have reported common side effects such as injection-site pain, fatigue, fever, and headache, which are generally mild and short-lived [6]. More serious side effects, including myocarditis, blood clotting disorders, and anaphylaxis, have been documented, although at much lower rates [7]. Gender differences in vaccine reactions have also been highlighted, with women tending to report more frequent and severe side effects, possibly due to immunological and hormonal factors [8]. Additionally, previous COVID-19 infection may influence vaccine responses, with some individuals experiencing stronger reactions due to immune priming [9]. However, most of these studies have examined general or mixed populations rather than medical students.

The rationale for this study stems from the need to fill this research gap and provide insights into the experiences and perceptions of COVID-19 vaccination among medical students in Bangladesh. As future healthcare providers, understanding their attitudes toward vaccination is crucial in addressing vaccine hesitancy and improving public health strategies. This study also seeks to examine how demographic factors, such as gender and previous COVID-19 infection, influence the side effects experienced by medical students. By focusing specifically on this group, the research aims to contribute valuable data that can guide vaccine rollout and communication strategies in Bangladesh and similar settings.

The primary objective of this study is to assess the types, frequency, and severity of side effects experienced by medical students in Bangladesh after receiving the COVID-19 vaccine. Furthermore, it aims to investigate the influence of demographic factors such as gender and previous COVID-19 infection on vaccine-related side effects. By exploring these factors, the study will also examine how medical students perceive the vaccines' safety and efficacy, and whether their experiences align with broader public health trends. The findings from this research will not only contribute to the academic understanding of vaccine side effects but will also inform public health policies, particularly in addressing concerns and promoting vaccine uptake among medical students and other healthcare professionals.

The findings from this study are particularly important for improving vaccine confidence, which is essential in ongoing efforts to control COVID-19 and prevent future pandemics. By understanding the specific concerns and experiences of medical students, this research can help shape targeted communication strategies that address vaccine hesitancy. Moreover, focusing on gender-based differences and prior COVID-19 infection will provide a more nuanced understanding of how different factors influence vaccine reactions, which can further aid in developing personalized public health approaches.

COVID-19 vaccination remains a key tool in the fight against the pandemic, but vaccine acceptance and safety concerns continue to pose challenges. This study aims to explore the experiences of medical students in Bangladesh, filling an important gap in the literature by investigating side effects and perceptions specific to this group. Given the central role of medical students in shaping future healthcare responses, understanding their experiences with COVID-19 vaccination will provide essential insights that can guide future public health policies and communication strategies aimed at improving vaccine uptake and confidence.

## Materials and methods

Study design

This descriptive, cross-sectional study was conducted among undergraduate medical students in Bangladesh between February 2021 and April 2022, coinciding with the nationwide COVID-19 vaccination program. An online survey was selected as the most feasible method due to pandemic-related restrictions on face-to-face data collection.

Study population and sample size

Participants were recruited using a convenience sampling approach through social media platforms (Facebook, WhatsApp, Messenger, Instagram, and LinkedIn), where the survey link was shared in medical student groups across different medical colleges. Based on group membership counts, the survey was estimated to have reached approximately 1,200 medical students. A total of 349 responses were received, of which 305 complete responses were included in the final analysis, yielding an estimated response rate of approximately 25.4% (305/1,200). Inclusion criteria included voluntary participation, being enrolled in a medical college in Bangladesh, receipt of at least one COVID-19 vaccine dose, and completion of the questionnaire. No incentives were offered to ensure voluntary participation and minimize response bias.

Study measures and data collection

Data were collected through a structured, self-administered electronic questionnaire designed in English, which is the standard medium of instruction in Bangladeshi medical schools. The questionnaire was adapted from previously validated tools used in studies assessing COVID-19 vaccine experiences [10-12]. It consisted of four sections: (1) socio-demographic characteristics: age, sex, academic phase, and area of residence; (2) medical history: comorbidities and previous COVID-19 infection; (3) vaccination details: vaccine type and number of doses received (dose 1, dose 2, and booster, where applicable); dose-wise analyses report denominators separately based on the number of respondents who received each dose; (4) post-vaccination experiences: including local (e.g., injection site pain and swelling) and systemic side effects (e.g., fever, myalgia, headache, rash, and anxiety).

Before participation, respondents were provided with an introductory section outlining the study’s purpose, confidentiality assurances, and the voluntary nature of participation. Informed electronic consent was obtained prior to completing the questionnaire. The full questionnaire is provided in the Appendices. Participants were instructed to report symptoms occurring within seven days after each vaccine dose. Multiple symptoms could be selected; therefore, total symptom counts exceed the number of respondents.

A formal pilot test of the questionnaire was not conducted due to rapid data collection requirements during the pandemic; however, the questionnaire was adapted from previously validated instruments, ensuring clarity and content relevance.

Ethical considerations

Ethical approval for this research was obtained from the Institutional Review Board (IRB) of Shaheed Ziaur Rahman Medical College, Bogura, Bangladesh (Approval number: 59.14.1020.135.000.25.0001.25.1303; date: 02/01/2021). The study adhered to the ethical principles outlined in the Declaration of Helsinki (2013 revision). Participation was voluntary, anonymity was maintained throughout, and participants retained the right to withdraw from the study at any stage without providing justification. No vaccination dates or identifiable personal information were collected.

Statistical analysis

Data were reviewed for completeness and coded in Microsoft Excel 2019 (Microsoft Corporation, Redmond, WA) before analysis in R version 4.3.2 (R Foundation for Statistical Computing, Vienna, Austria). Descriptive statistics were used to summarize participant characteristics and vaccination experiences. Categorical variables were presented as frequencies and percentages, and continuous variables as mean (standard deviation). Dose-wise analyses used separate denominators based on the number of respondents who received each dose. “Any side effect” was defined as reporting ≥1 local or systemic symptom after a specific dose; participants could select multiple symptoms, therefore symptom totals exceed the number of respondents.

Associations between categorical variables (e.g., vaccine type, sex, and presence of side effects) were examined using Pearson’s chi-square test (χ²) or Fisher’s exact test when expected cell counts were <5. To identify independent predictors of post-vaccination side effects, multivariable Firth logistic regression was applied to account for sparse cell counts and potential data separation. The Firth regression applies a penalized likelihood approach that reduces small-sample bias and produces more reliable parameter estimates when certain vaccine-dose subgroups contain very few observations. Covariates entered into the model included sex, age, vaccine type, prior COVID-19 infection, presence of comorbidities, academic phase, and area of residence. Reference categories were specified as male sex, no prior COVID-19 infection, no comorbidity, first vaccine dose, and urban residence. Missing data were minimal and handled using complete-case analysis. Statistical significance was defined as p < 0.05.

Dose-specific and vaccine-specific analyses were conducted using only participants who received that dose and that vaccine. Denominators, therefore, vary across tables (e.g., 125 Sinopharm dose 1 recipients and 22 Pfizer-BioNTech dose 3 recipients).

Patient and public involvement

No patients or members of the public were involved in the design, implementation, or dissemination of this study, as it focused exclusively on medical students.

## Results

Participant characteristics

A total of 305 medical students participated in the study. The mean age was 21.6 years (SD = 1.43; range = 18-27). Females represented 196 participants (64.3%) of the total sample. Participants were distributed across all academic phases, with the fourth phase most represented (91 participants, 29.8%). Approximately half of the participants resided in urban areas (152, 49.8%) (Table 1).

**Table 1:** Demographic characteristics of the participants.

Characteristic	Number (n)	Percentage (%)
Male	109	35.7
Female	196	64.3
1st year students	80	26.2
2nd year students	67	22
3rd year students	67	22
4th year students	91	29.8
Rural	56	18.4
Sub-urban	97	31.8
Urban	152	49.8

Vaccine types

**Figure 1:**
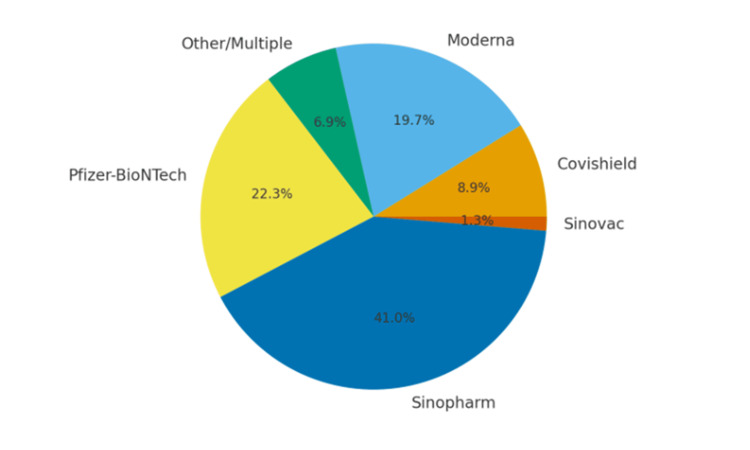
Distribution of COVID-19 vaccine types received by participants (n = 305).

The types of vaccines received and dose uptake are shown in Figure 1. Sinopharm (125 participants, 41.0%) and Pfizer-BioNTech (68 participants, 22.3%) were the most frequently reported vaccines, followed by Moderna (60 participants, 19.7%).

First- and second-dose uptake exceeded 90%, with 293 participants (96.1%) and 286 participants (93.8%), respectively, whereas 191 participants (62.6%) received a third dose. Table 2 summarizes the number of participants who received each vaccine type at each dose, which served as denominators for dose- and vaccine-specific analyses.

**Table 2:** Number of participants receiving each vaccine type by dose (denominators for dose- and vaccine-specific analyses).

Vaccine type	Dose 1 (n)	Dose 2 (n)	Dose 3 (n)
Sinopharm	125	125	99
Pfizer-BioNTech	67	64	22
Moderna	59	57	41
Covishield (AstraZeneca)	27	27	20
Sinovac	4	4	3
Mixed/multiple vaccine types	3	3	2
Forgotten/unspecified	1	0	0

Side effects

The prevalence of self-reported post-vaccination side effects is presented in Table 3. Overall, 276 participants (90.5%) experienced at least one side effect. Side effects were most frequently reported after the first dose (270, 88.5%), followed by the second dose (243, 79.7%), and were least common after the third dose (128, 42.0%).

**Table 3:** Prevalence of side effects following vaccination. Note: Denominator varies by dose and vaccine type.

Outcome	Number (n)	Percentage (%)
Any side effect	276	90.5
After 1st dose	270	88.5
After 2nd dose	243	79.7
After 3rd dose	128	42

The distribution of specific adverse events is presented in Table 4. Participants were allowed to select more than one symptom; therefore, the total number of reported events exceeds the number of respondents (n = 305), and percentages are calculated based on the total number of symptom reports rather than individuals. Local pain was the most frequently reported event (508 reports, 41.2%), followed by fever (184, 14.9%), allergy/rash/itch (114, 9.2%), headache (108, 8.8%), and myalgia/body ache (103, 8.3%). Less common symptoms included anxiety or depressed mood (70, 5.7%), alopecia (45, 3.6%), and loss of appetite (29, 2.4%). Rare events, such as blood pressure changes (2, 0.2%), ocular symptoms (2, 0.2%), and dizziness or syncope (1, 0.1%), were infrequently reported.

**Table 4:** Frequency distribution of specific adverse events. Note: Participants could report more than one adverse event; therefore, counts and percentages represent symptom reports, not the number of individuals.

Adverse event	Frequency (n)	Percentage (%)
Local pain	508	41.2
Fever	184	14.9
Allergy/rash/itch	114	9.2
Headache	108	8.8
Myalgia/body ache	103	8.3
Anxiety/depressed mood	70	5.7
Other/unspecified	55	4.5
Alopecia/hair loss	45	3.6
Loss of appetite	29	2.4
Swelling (local)	13	1.1
Blood pressure change	2	0.2
Ocular symptoms	2	0.2
Dizziness/syncope	1	0.1

Correlation analysis

Associations between participant characteristics and reported side effects are summarized in Table 5. Vaccine type showed significant associations with side effects overall and at each dose. Sex was significantly associated with side effects following the second dose. Fisher’s exact tests are reported where expected cell counts were small.

**Table 5:** Associations between participant characteristics and side effects. χ² = Chi‑square statistic; df = degrees of freedom. Fisher’s exact test was applied where expected counts were small. Note: Denominator varies by dose and vaccine type.

Comparison	Chi-square (χ²)	Degrees of freedom (df)	P-value (Chi-square)	P-value (Fisher’s exact)
Sex × Any side effect	1.6318	1	0.2014	
Vaccine type × Any side effect	88.911	5	<0.0001	8.21E-11
Comorbidity × Any side effect	5.8668	9	0.7532	0.5712
COVID-19 screening × Any side effect	2.9749	3	0.3955	0.5872
Sex × Side effects (1st dose)	0.5575	1	0.4553	
Sex × Side effects (2nd dose)	4.7525	1	0.0293	
Sex × Side effects (3rd dose)	1.6121	1	0.2042	
Vaccine × Side effects (1st dose)	71.37	5	5.32E-14	
Vaccine × Side effects (2nd dose)	40.513	5	1.18E-07	
Vaccine × Side effects (3rd dose)	20.902	5	0.00085	

Significant predictors of side effects identified through multivariable Firth logistic regression are shown in Table 6. Receipt of other or mixed vaccines (vs. Moderna) was associated with lower odds of experiencing side effects overall and after the first dose. Female sex was associated with increased odds of side effects after the second dose. For the third dose, receipt of Pfizer-BioNTech (vs. Moderna) was associated with lower odds of reporting side effects.

**Table 6:** Significant predictors of side effects in multivariable Firth logistic regression. OR: odds ratio; C: confidence interval. Vaccine reference = Moderna; Sex reference = Male.

Predictor (reference)	Outcome	Odds ratio (OR)	95% Confidence interval (CI)	P-value
Other/Multiple vs. Moderna	Any dose	0.29	0.091–0.811	0.0177
Other/Multiple vs. Moderna	1st dose	0.363	0.124–0.969	0.0429
Female vs. Male	2nd dose	1.853	1.054–3.256	0.0324
Pfizer-BioNTech vs. Moderna	3rd dose	0.446	0.209–0.938	0.0331

## Discussion

In this cross-sectional study of 305 medical students in Bangladesh, a strikingly high proportion of participants (90.5%) reported at least one post-vaccination side effect. Local reactions and systemic symptoms were common, with local pain (41.2%) emerging as the predominant complaint, followed by fever (14.9%), allergy or rash (9.2%), headache (8.8%), and myalgia or body ache (8.3%). Psychological and dermatological manifestations such as anxiety or depressed mood (5.7%) and alopecia or hair loss (3.6%) were also noted, although less frequently. By contrast, potentially serious or rare events, including blood pressure fluctuations (0.2%), ocular disturbances (0.2%), and dizziness or syncope (0.1%), were seldom observed. Side effects were reported more often after the first dose (88.5%) compared with the second (79.7%) and third (42.0%) doses, suggesting a decreasing trend with successive administrations. The analysis further highlighted important demographic and vaccine-related associations: female students were significantly more likely than males to report side effects after the second dose (χ² = 4.75, p = 0.029), while vaccine type was strongly associated with adverse event profiles across all doses (overall χ² = 88.9, df = 5, p < 0.001). Multivariable regression confirmed that sex and vaccine type remained independent predictors, with female sex associated with increased odds of side effects after the second dose (OR: 1.85, 95% CI: 1.05-3.26) and Pfizer-BioNTech recipients less likely than Moderna recipients to report adverse events after the third dose (OR: 0.45, 95% CI: 0.21-0.94).

Beyond the high prevalence of expected local and systemic reactions, the emergence of less common outcomes such as psychological symptoms (5.7%) and alopecia (3.6%) in our cohort deserves attention. Although these findings were infrequent, their occurrence among young medical students raises questions regarding underlying mechanisms. Some authors have suggested that vaccine-associated anxiety is often secondary to psychosocial stressors, risk perception, and the heightened vigilance surrounding COVID-19, rather than a direct pharmacological effect of the vaccine [13]. Similarly, alopecia has been sporadically reported following both infection and vaccination, with proposed pathways including stress-induced telogen effluvium and immune-mediated dysregulation [14,15]. While causality cannot be established from observational data, documenting such outcomes is important to provide a comprehensive account of vaccine experiences, particularly in younger populations where psychological well-being is a key concern.

A dose-dependent decline in self-reported reactogenicity in our cohort, i.e., highest after the first dose (88.5%) and falling after the second (79.7%) and third (42.0%) doses, merits comment against broader evidence. App-based surveillance in the UK reported platform-specific patterns, with systemic symptoms more frequent after the second than the first dose for mRNA vaccines but attenuated after subsequent exposures in many recipients [16]. Large US safety datasets similarly indicate that local reactions remain common yet generally mild after additional doses, while systemic symptoms after boosters are typically transient and comparable to or lower than those observed after the primary series [17,18]. Randomized and observational studies of third-dose schedules corroborate this attenuation, describing acceptable reactogenicity profiles across platforms [19,20]. By contrast, inactivated vaccines have consistently shown a lower systemic reactogenicity burden across doses than mRNA products [21,22], which may partly explain the downward trajectory observed here, given the high uptake of Sinopharm in our sample. Clinical trial evidence for ChAdOx1, BNT162b2, and mRNA-1273 indicates that systemic reactogenicity can be more prominent following the second dose, yet adverse events remain transient and of mild-to-moderate intensity in the vast majority of cases [5-7].

The observation that female students reported significantly higher odds of side effects after the second dose is consistent with a well-established gender disparity in vaccine reactogenicity. Multiple studies across different populations have shown that women are more likely to report both local and systemic adverse events following COVID-19 vaccination [23-25]. Biological explanations include stronger innate and adaptive immune responses influenced by sex hormones and X-linked immune-related genes [26,27]. Beyond physiology, gendered patterns in health awareness and care-seeking behavior may amplify reporting differences, with women generally more attuned to and willing to disclose symptoms than men [28]. A cross-sectional study of healthcare workers in the Czech Republic found a significantly higher prevalence of post-vaccination reactions in women, particularly systemic symptoms such as fatigue and headache [23]. Similarly, data from Saudi Arabia and the United States confirmed this pattern, noting that female sex was a predictor of higher reactogenicity regardless of vaccine platform [24,25]. Such consistency across settings underscores the importance of recognizing sex-based differences, not only in clinical communication about expected vaccine responses but also in tailoring future surveillance and pharmacovigilance strategies.

The variation in adverse event profiles observed across vaccine types in this study also reflects global evidence of platform-specific differences. Recipients of inactivated vaccines, such as Sinopharm, have generally reported fewer systemic side effects compared with mRNA-based vaccines, although local injection site pain remains common [29,30]. A rapid review of vaccine safety confirmed this gradient, noting that systemic reactogenicity, particularly fever, chills, and fatigue, was most frequent with mRNA platforms, intermediate with adenoviral-vectored vaccines, and lowest with inactivated products [31]. Data from multi-country trials further demonstrate that although mRNA vaccines elicit higher rates of transient systemic symptoms, they provide strong and durable protection against severe COVID-19 outcomes [5,7,32]. Our regression analysis suggested that Moderna recipients were more likely than Pfizer recipients to report adverse events after the third dose, a finding echoed in studies attributing such differences to formulation strength and dose size [33]. Understanding these comparative profiles is valuable not only for clinical counseling but also for guiding national vaccine strategies, particularly in contexts where multiple platforms are concurrently deployed.

The implications of these findings extend beyond individual experiences and are particularly important in the context of medical students as future healthcare providers. Vaccine confidence among medical trainees not only influences their own uptake but also shapes their readiness to recommend vaccination to patients and the wider public. Previous studies have shown that medical and nursing students who experience minimal side effects are more likely to perceive vaccines as safe and to advocate for them in their professional roles [34,35]. Conversely, negative personal experiences or heightened concern about adverse events can foster hesitancy, undermining their role as credible public health ambassadors [36]. A pooled analysis across seven countries reported that vaccine acceptance among healthcare students was strongly associated with perceived safety, trust in scientific authorities, and prior positive experiences with immunization [37]. In settings such as Bangladesh, where misinformation and vaccine hesitancy persist, strengthening medical students’ confidence through transparent communication about side effects and evidence-based reassurance is critical. Embedding vaccination education into medical curricula, alongside training in risk communication, may empower this group to counter misinformation effectively and promote vaccine uptake within their communities.

This study has several limitations that should be considered when interpreting the findings. First, the use of convenience sampling and reliance on self-reported data may introduce recall and reporting bias, potentially overestimating or underestimating side-effect prevalence. Similar challenges have been noted in other cross-sectional surveys of vaccine experiences, where symptom reporting varied with participant awareness and survey timing [38,39]. Because recruitment was conducted via social media, students who were more digitally connected, urban-based, or motivated to share their experiences may have been more likely to participate; this may inflate the reporting of adverse events. Although responses were obtained from students representing 25 medical colleges across Bangladesh, representativeness across all institutions cannot be ensured. Second, the study did not collect vaccination dates, preventing analysis of the time interval between vaccination and symptom onset, and the questionnaire was not pilot-tested due to the rapid data collection context. Third, the study did not capture the duration or severity of adverse events, limiting comparability with clinical trial data where these parameters are systematically assessed. Additionally, the cross-sectional design precludes causal inference, as associations between demographic factors and side effects cannot establish temporality. Prior research has highlighted the value of longitudinal follow-up in identifying delayed or persistent effects, which was not feasible here [40]. Finally, the study population comprised only medical students, a group that may differ from the general population in terms of health literacy, risk perception, and reporting behavior. While this focus provides insights into a key future healthcare workforce, broader studies, including other professional groups and community samples, are necessary for a more representative picture. Additionally, data were collected across multiple vaccination phases (2021-2022), during which vaccine availability and circulating variants differed, introducing potential temporal heterogeneity in reactogenicity. Finally, psychological symptoms such as anxiety or low mood may reflect pandemic-related stress rather than direct vaccine effects, and should be interpreted cautiously.

This study contributes to the growing body of evidence on COVID-19 vaccine safety by documenting the prevalence and determinants of side effects among medical students in Bangladesh. The findings reaffirm the generally benign nature of post-vaccination symptoms while highlighting gender-based differences, vaccine-type variability, and the occurrence of less common outcomes such as psychological and dermatological manifestations. Importantly, the study underscores the need to address vaccine concerns within medical education, as medical students’ attitudes and experiences will influence their future role as public health advocates. Strengthening vaccine confidence through transparent communication and integration of vaccine literacy into curricula could not only enhance uptake in this group but also foster broader trust in immunization programs. As the global community prepares for future pandemics, lessons from the COVID-19 vaccination campaign, particularly in resource-limited settings, will remain highly relevant for sustaining vaccine confidence and ensuring effective responses to emerging infectious threats [41].

## Conclusions

COVID-19 vaccination among Bangladeshi medical students was generally safe, with most reported side effects being mild and self-limiting. These findings underline the importance of sustaining transparent communication about vaccine safety and improving vaccine-related training within medical curricula to equip future physicians with confidence in counseling patients. Moreover, it may help clinicians and medical educators reassure patients by emphasizing that post-vaccination symptoms are generally mild and transient. Future research should expand to broader populations and incorporate longitudinal follow-up to better understand the duration and clinical relevance of less common post-vaccination outcomes.

## Appendices

Survey questionnaire

**Table 7:** Survey questionnaire.

1	The objective of this study is to assess the post-vaccination status & better understand the experiences. The anonymity and the confidentiality of the information will be strictly maintained. Do you wish to voluntarily participate?
	• Yes
	• No
2	Age
	………………..
	(Example 01/07/2002)
3	Sex
	• Male
	• Female
4	Education
	• 1st Phase MBBS
	• 2nd Phase MBBS
	• 3rd Phase MBBS
	• 4th Phase MBBS
5	Division
	• Dhaka
	• Chittagong
	• Sylhet
	• Rajshahi
	• Barishal
	• Khulna
	• Rangpur
	• Mymensingh
6	Residence
	• Rural
	• Sub-urban
	• Urban
7	Other comorbidities
	• Allergy
	• Asthma
	• Diabetes
	• Heart disease
	• Hypertension
	• Thyroid disease
	• Tuberculosis
	• None
	• Other:……..
8	COVID-19 screening
	• Tested negative
	• Tested positive
	• I didn’t test, but I had all the symptoms
	• I didn’t test because I didn’t have any symptoms
9	Vaccination status
	• Yes
	• No
10	1st dose taken
	• Yes
	• No
11	2nd dose taken
	• Yes
	• No
	3rd dose taken
12	• Yes
	• No
13	Vaccine name
	• Moderna
	• Covishield
	• Sinopharm
	• Pfizer-BioNTech
	• Sinovac
	• Other: ………
14	After 1st dose
	Check all that apply
	• Local pain
	• Body ache for more than 24 hours
	• Headache
	• Fever
	• Loss of appetite
	• Rash
	• Allergy
	• Anxiety
	• Depression
	• High blood pressure
	• Low blood pressure
	• Itching
	• Alopecia or hair loss
	• Acne
	• Other:…….
15	After 2nd dose
	Check all that apply
	• Local pain
	• Body ache for more than 24 hours
	• Headache
	• Fever
	• Loss of appetite
	• Rash
	• Allergy
	• Anxiety
	• Depression
	• High blood pressure
	• Low blood pressure
	• Itching
	• Alopecia or hair loss
	• Acne
	• Other:…….
16	After 3rd dose
	Check all that apply
	• Local pain
	• Body ache for more than 24 hours
	• Headache
	• Fever
	• Loss of appetite
	• Rash
	• Allergy
	• Anxiety
	• Depression
	• High blood pressure
	• Low blood pressure
	• Itching
	• Alopecia or hair loss
	• Acne
	• Other:…….
17	Admitted to the hospital after taking the vaccines
	• Yes
	• No
